# Editorial: Positive Organizational Interventions: Contemporary Theories, Approaches and Applications

**DOI:** 10.3389/fpsyg.2020.607053

**Published:** 2020-11-17

**Authors:** Llewellyn E. van Zyl, Sebastiaan Rothmann

**Affiliations:** ^1^Department of Industrial Engineering, University of Eindhoven, Eindhoven, Netherlands; ^2^Optentia Research Focus Area, North-West University, Vaal Triangle Campus (VTC), Vanderbijlpark, South Africa; ^3^Department of Human Resource Management, University of Twente, Enschede, Netherlands; ^4^Department of Social Psychology, Institut für Psychologie, Goethe University, Frankfurt am Main, Germany

**Keywords:** positive organizational interventions, positive psychological interventions, strengths based development, positive psychological coaching, talent management

## Introduction

Positive Organizational Interventions (POIs) have emerged as popular mechanisms to facilitate the personal/professional development and well-being of individuals as well as optimizing the growth potential of organizations (van Zyl and Rothmann, 2019a). These interventions draw from positive psychological principles, processes, and practices in order to produce positive outcomes for the individual (e.g., work engagement), the team (e.g., collaboration, team flow) and ultimately, the organization (e.g., innovative work behaviors) (Costantini et al., 2019). Through the optimization, utilization and application of an entity's strengths, POIs are not aimed at fixing what is proverbially “wrong,” or correcting deviant behaviors, but rather focus on enhancing what is already working well (van Zyl and Rothmann, 2019b). This positive approach toward individual and organizational development has gained mass-appeal within the popular psychological press circuit (“Pop Psych”) and is readily implemented within organizational contexts (Haberlin, 2019; van Zyl et al.).

Pop Psych has increased the visibility of POIs within the broader population through providing access to scientific content in an easily digestible format (Seligman, 2012). Pop Psych authors promise “*ten scientifically proven ways*” or “*five easy evidence-based practices*” to build flourishing organizations, optimal functioning teams, positive leaders and thriving employees through translating “scientifically proven interventions” into consumer-friendly terms (Ausch, 2016). However, the scientific merits of these portrayals within the Pop Psych press are questionable as authors miscommunicate findings, provide erroneous “summaries” of papers, and fundamentally alter both the context of- and POIs protocols published within the scientific literature (Ferguson, 2015, 2019). Therefore, when these interventions are implemented within practice, they rarely yield functional returns and do not deliver on their promises (Ausch, 2016). This in turn has a negative impact on the perceptive value of POIs within both the public domain and the broader discipline of psychology.

Scientists within the broader discipline of psychology have also questioned the effectiveness of POIs and shown to be critical of the underlying paradigm (i.e., positive psychology) (c.f. Brown et al., 2014; Friedman and Brown, 2018; Wong and Roy, 2018; Trask-Kerr et al., 2019). Academic authors have highlighted that POIs produce mixed results outside of clinical contexts (Bolier et al., 2013; Roll et al., 2019), that intervention effects are rarely replicable (Mongrain and Anselmo-Matthews, 2012; Khanna and Singh, 2019) or sustained (Turnes et al., 2020; van Zyl et al., 2020), that reported effect sizes are usually small (Bolier et al., 2013) and that the effectiveness of POIs are highly dependent upon contextual factors (Parks and Schueller, 2014; Wong and Roy, 2018).

Contemporary literature suggests the lack of effectiveness of POIs is a function of a plethora of factors ranging from insufficient duration of the intervention, inappropriate dissemination methods, inadequate consideration of cultural and contextual factors, to a failure to build interventions around validated theoretical models, poor measurement strategies and person-activity misfit (Stander and van Zyl, 2019; van Zyl et al., 2019). In essence, the problem stems from poor POI design, implementation and evaluation methods which is exasperated by a lack extensive POI intervention protocols and methodologies within the academic literature. In those instances where POIs have shown to be effective, intervention content is usually condensed into a single paragraph in the methods' section or removed in its entirety in the final manuscript. This severely limits or deludes its potential transferability into practice.

In order to address these challenges, practitioners and researchers need to develop a shared understanding as to how POIs need to be designed, how content needs to be aligned to the strengths of participants, how to effectively evaluate such and finally how to maintain the positive effects over time. Similarly, clear intervention protocols need to be established, practice friendly process models need to be provided and the models on which interventions are built needs to be expanded. As such, the purpose of this Research Topic and e-book was to address these challenges through curating innovative theoretical and empirical POI research relating to modern intervention designs, methodologies, models, content, and evaluation methods.

## Structure and Contribution of this Special Issue

The primary aim of this manuscript was to collate a collection of contemporary approaches toward the development, implementation and evaluation of POIs which could easily be translated into practical, viable instruments for others to employ. The 12 manuscripts in this special issue, summarized in Table 1, are classified into four sections:

1. *Empirically validated positive organizational intervention strategies*. Here the focus is on determining the effectiveness of POIs and to present readers with intervention protocols.
2. *Empirical models for positive organizational interventions*. In this section, authors aimed to provide a proverbial “roadmap” on which POIs can be built, and to show how adopting a certain type of intervention approach may impact organizational outcomes.
3. *Positive organizational intervention strategies and frameworks*. Here the focus was on providing broad practice friendly POI strategies and frameworks.
4. *Online POI design principles*. In this section, the authors attempted to provide an overview of important components to consider when designing engaging online POIs.

**Table 1:** Summary of the contributions to this special topic.

**No**	**Author**	**Title of contribution**	**Main objective**
**SECTION 1: EMPIRICALLY VALIDATED POSITIVE PSYCHOLOGICAL INTERVENTION STRATEGIES**			
1	Paver et al.	The implementation and evaluation of the South African adaptation of the JOBS program	The purpose of this paper was to evaluate the effectiveness of a POI aimed at enhancing the job-related search behaviors of unemployed individuals within the South African context. The JOBS program aimed to enhance the self-efficacy, amotivation and self-esteem of job-seekers.
2	Lock et al.	Feasibility and process evaluation of a need-supportive physical activity program in aged-care workers: the Activity for well-being project	In this paper, the authors implemented a mixed-methods process evaluation and feasibility study for a need-supportive physical activity program that was piloted in a single-group pre-post study. The piloted program was designed to support participant needs of autonomy, competence and relatedness through evidence-based and theory-informed behavior change strategies including a Motivational Interviewing style appointment, education on self-management tools, and Self-Determined modes of regulating physical activity intensity. The program aimed to positively impact physical activity behavior, psychological well-being and associated motivational processes.
3	Kreemers et al.	Testing a self-compassion intervention among job seekers: self-compassion beneficially impacts affect through reduced self-criticism	In this paper, the authors examined whether state self-compassion can be increased among job seekers through writing exercises in which job seekers are instructed to reflect with self-compassion on their negative job search experiences. Further, they wanted to determine if a self-compassion intervention benefited job seekers' affective responses, through reducing self-criticism.
4	Peeters et al.	Positive psychological micro-interventions to improve the work–family interface: use your resources and count your blessings	The authors aimed to investigate the effectiveness of two micro-interventions which aimed to improve the work-family interface. One intervention focused on aiding individuals to optimize the use of their resources and the other focused on becoming consciously aware of one's “blessings.”
5	Hulshof et al.	Providing services during times of change: can employees maintain their levels of empowerment, work engagement and service quality through a job crafting intervention?	This paper focused on how a job crafting training programme could aid organizations to maintain empowerment, work engagement and performance during times of organizational change.
6	Peláez-Zuberbuhler et al.	Coaching-based leadership intervention program: a controlled trial study	The authors of this paper implemented and evaluated a coaching-based leadership intervention program comprised of training and three coaching sessions. The results showed that the intervention was successful in enhancing one's coaching-based leadership skills, psychological capital, work engagement and performance.
7	Bergsma et al.	Will happiness-trainings make us happier? A research synthesis using an online findings-archive	The authors investigated the effectiveness of happiness trainings using an online research repository. Specifically, they provided an overview of the techniques that can be used to enhance happiness, the duration of how long happiness trainings effects the happiness of participants, and what kind of individual could benefit from happiness trainings.
**SECTION 2: EMPIRICAL MODELS FOR POSITIVE ORGANIZATIONAL INTERVENTIONS**			
8	Haider et al.	A three-wave longitudinal study of moderated mediation between high-performance work systems and employee job satisfaction: the role of relational coordination and peer justice climate	The authors provided an extensive exploration for an empirical model on which to build positive organizational level interventions aimed at enhancing job satisfaction. Through a three-wave longitudinal design, the authors found that organizations should foster practices that help foster relational coordination between employees in order to increase their job satisfaction. Further, managers should focus on optimizing rewards/recognition systems, performance management and meetings in order to create a positive and supportive work environment.
9	van Woerkom and Kroon	The effect of strengths-based performance appraisal on perceived supervisor support and the motivation to improve performance	The authors examined the effect of strengths-based performance appraisals on the relationship between the support supervisors provide and its impact on motivation to enhance performance. The findings indicate that a focus on strengths in the performance appraisal may boost employees' optimism regarding future successes, which is especially important to safeguard a supportive relationship with the supervisor when the performance rating is relatively low.
**SECTION 3: POSITIVE ORGANIZATIONAL INTERVENTION STRATEGIES AND FRAMEWORKS**			
10	van Zyl et al.	Positive psychological coaching definitions and models: a systematic literature review	The authors attempted to consolidate the literature on positive psychological coaching in order to provide an integrated definition and practice-orientated model.
11	Jonker et al.	An intervention framework to facilitate psychological trauma management (PTMP) in high-risk occupations	This study provided a qualitatively explored the experiences of PTMPs from the perspective of employees working in three high-risk occupations. The study explored the experiences of the participants in order to compile a framework that supports and improves the productivity and well-being of employees affected by work-related trauma.
**SECTION 4: ONLINE POSITIVE ORGANIZATIONAL INTERVENTION DESIGN PRINCIPLES**			
12	Kelders et al.	The concept and components of engagement in different domains applied to ehealth: a systematic scoping review	The aim of this study was to determine what constitutes “engagement” in order to effectively translate such into effective e-Health designs. The authors attempted (a) to investigate in which domains engagement features, (b) to determine what constitutes engagement in these different fields, and (c) to determine whether there are any common components that seem to be important.

The papers in each of these respective sections advances our understanding as to what constitutes a POI, as well as how such should effectively be designed and implemented. On a meta-level, this special issue highlights the following:

1. POIs largely involve a structured set of intentional developmental initiatives that are initiated by an organization, that are built upon the positive psychology paradigm with the specific aim to promote positive states, traits, behaviors and to facilitate a positive organizational climate and culture.
2. POIs could take the form of *self-administered intentional activities* (e.g., counting one's blessings), *group-based development initiatives* (e.g., happiness trainings), *organizational level interventions* (e.g., strengths-based performance management) and *strengths- or positive coaching*.
3. POIs that do not produce the desired effects are largely the result of poor empirical models underpinning interventions, interventions focusing on enhancing outcomes rather than specifically targeted mediators/moderators, poor intervention design and unreliable measuring instruments, as well as person-activity misfit and when basic behavioral change models are ignored.
4. In terms of POI design, the focus should be placed on the duration of the intervention, participants intention-to-treat, appropriate dissemination methods or tools should be employed, and the culture and context of participants need to be taken into consideration.
5. Effective POIs focus on aligning the features of the intervention and the methodology of its dissemination, to the personal features of the participants and
6. Participants and facilitators need to be debriefed after the completion of the intervention.

## Guidelines For Designing Positive Organizational Interventions

**Figure 1:**
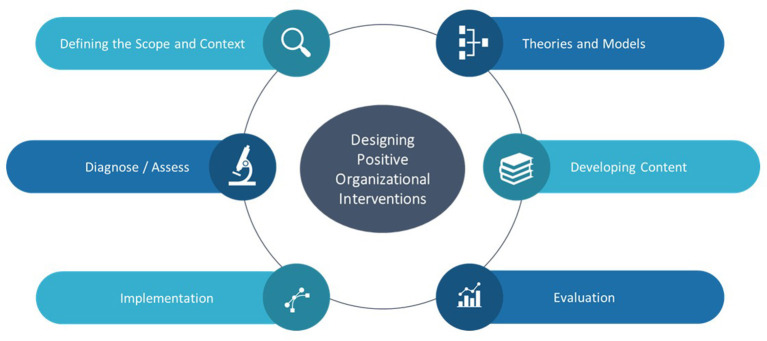
Elements to consider when designing POIs.

This special issue further highlights that the effectiveness of POIs is fundamentally a function of its intentional design. Although various attempts have been made to provide structured guidelines on designing POIs and Positive Psychological Interventions (van Zyl et al., 2019), no clearly validated frameworks or protocols for such exist. This special issue highlights six elements of designing impactful POIs (see Figure 1).

When designing POIs, researchers and practitioners should consider the following:

- *Defining the scope and context of the intervention*. Here the focus is on understanding the nature of the underlying problem within the population and determining the way to effectively address and approach such. Practitioners should first conduct a needs analyses to determine the underlying needs of participants. These needs need to be reformulated/categorized into specific positive states, -traits and behaviors that need to be targeted by the intervention. Practitioners should therefore carefully consider the appropriateness and relevance of these factors/constructs for the specific context (Alexandrova, 2017) as such would influence the effectiveness of the intervention. The specific context in which the intervention is to take place, plays a major role in both the adoption of- and adherence to intervention content and should therefore be an essential element of investigation during the scoping/panning process. In essence, the purpose of the intervention, the target population group, the level of the intervention, the needs/context of participants and the specific positive factors to be targeted needs to be considered in this phase.
- *Building interventions around positive psychological theories and models*. Effective interventions start with a clarifying a core positive outcome to be achieved and be aligned to an appropriate positive psychological theory or model. This model is used to provide a roadmap on which the developmental practices can be built. Here one needs to ensure that the positive psychological outcome to be achieved is aligned to the nature, scope and treatment trajectory. Further an appropriate behavioral change model needs to be selected to understand the facilitators and barriers that impacts changes in behaviors. These models should clearly articulate both the positive states/traits/behaviors to be targeted as well as the specific repertoire of skills, capabilities and resources required to enhance such (Oades et al., 2020).
- *Validated diagnostic frameworks and assessment techniques/tools need to be employed*. Validated positive psychological assessment techniques/instruments need to be used to assess the core components of the theoretical model being tested. The entire measurement/diagnostic model needs to be tested in the beginning and end of the intervention. Practitioners should further assess participants' intention to treat and willingness to participate. Clear goals for the intervention and an appropriate evaluation strategy needs to be developed before the start of the intervention.
- *Developing intervention content aligned to the empirical model and that “fits” the participant*. Intervention content needs to be aligned to the components of the empirical model, with functional developmental activities designed to enhance the specific state/behaviors being targeted. Intervention activities should be designed to develop specifics skills and capabilities within real-world environments; actively considering the contextual resources and constraints of participants. Intervention content should be novel and not repetitive, but also be challenging, and provide opportunities for autonomous practice. Intervention should also be designed around the utilization of individual strengths, and optimal usage of currently available personal/social resources in order to enhance the effectiveness and sustainability of its positive effects over time. Further, attempts need to be made to align intervention content to personal characteristic of participants to enhance intervention adherence. Specific focus needs to be placed on ensuring both person-intervention fit, as well as context-intervention fit to increase adherence and engagement. Finally, the means through which the content is to be delivered and the time frame for delivery needs to be defined. The mode of delivery needs to be aligned to the personal characteristics and capabilities of participants. All these elements need to culminate in the development of a structural intervention protocol.
- *Interventions need to be structurally implemented and appropriately managed*. The effectiveness of an intervention is not only dependent upon the design, but also in how it is executed. Practitioners should ensure adherence to the intervention protocol, and only intervene if participants are showing adverse reactions to the content. Practitioners should actively monitor both the effect of the intervention and participant engagement/adherence.
- *POIs need to be appropriately evaluated*. Changes in the positive psychological outcome (dependent variable) need to be actively monitored. Both quantitative and qualitative assessment measures need to be employed. Direct feedback during the intervention process need to be solicited from participants.

## Conclusion

Despite significant advancements in the field of applied positive psychology, and the popularization of a “positive approach toward people and organizational development” in practice, intervention research still seems to be in its infancy. We therefore hope that this special issue will provide the reader with some context as to our current understanding of POIs and stimulate future researchers to further investigate how such can be optimally designed, implemented, and evaluated.

## Author Contributions

Both authors listed have made a substantial, direct and intellectual contribution to the work, and approved it for publication.

## Conflict of Interest

The authors declare that the research was conducted in the absence of any commercial or financial relationships that could be construed as a potential conflict of interest.
